# Osteomalacia and vitamin D deficiency in a psychiatric rehabilitation unit: case report and survey

**DOI:** 10.1186/1756-0500-2-82

**Published:** 2009-05-09

**Authors:** Rudolf N Cardinal, Carol A Gregory

**Affiliations:** 1 Behavioural and Clinical Neurosciences Institute and Department of Psychiatry, University of Cambridge, Addenbrooke's Hospital, Hills Road, Cambridge, CB2 0QQ, UK; 2 Cambridgeshire and Peterborough NHS Foundation Trust, Fulbourn Hospital, Cambridge Road, Cambridge, CB21 5EF, UK

## Abstract

**Background:**

Vitamin D deficiency is common and predisposes to many serious diseases, yet often goes unrecognized.

**Findings:**

We describe a case of severe vitamin D deficiency with osteomalacia in a patient resident in a psychiatric hospital for more than 35 years, and discuss causes and complications. We assayed the serum 25-hydroxyvitamin D levels of all patients under our care on one old-age psychiatry rehabilitation unit. Ten of twelve (83%) of patients had vitamin D deficiency, and 92% had suboptimal vitamin D levels. Vitamin D status was strongly predicted by dietary supplementation. Of those not on vitamin D supplements, 100% had vitamin D deficiency, with vitamin D levels significantly below those of historical controls. Age, sex, and duration of admission did not predict vitamin D status in this group.

**Conclusion:**

We advocate vitamin D screening in all patients admitted to psychogeriatric units, and discuss treatment options given the current problems affecting high-dose vitamin D supply to the United Kingdom.

## Background

Vitamin D deficiency is so common as to represent a major public health problem, particularly in the elderly [1], yet it often goes unrecognized. We report on a patient who suffered complications from osteomalacia that had gone unrecognized for some time, and report a very high prevalence of vitamin D deficiency amongst inpatients on a psychiatric rehabilitation ward. We review the basics of vitamin D metabolism, and the causes and clinical features of osteomalacia. We offer practical suggestions for psychiatrists for the treatment of simple vitamin D deficiency, which in the United Kingdom is presently complicated by problems affecting the supply of high-dose vitamin D.

## Methods

We provide a case report of a patient with severe vitamin D deficiency, illustrating its clinical presentation. Her case is unusual because she has been an inpatient for more than 35 years, meaning that her diet and activity have in large part been under hospital supervision, and that she has had frequent surveillance of serum biochemical indices relevant to osteomalacia. We measured the vitamin D status (serum 25-hydroxyvitamin D level) in April–May 2008 of all patients under our care on the same psychogeriatric rehabilitation ward (*n *= 12).

Serum 25-hydroxyvitamin D was measured by radioimmunoassay (Immunodiagnostic Systems Ltd, Boldon, UK) in a UK Clinical Pathology Accreditation (CPA) approved laboratory [2]. The assay under-recovers plant-derived 25-hydroxyvitamin D_2 _but not 25-hydroxyvitamin D_3 _(for assay specifications see [2] and the manufacturer's data at ). Local control values were derived from repeated measurements over one year of 25-hydroxyvitamin D in 96 healthy Caucasian East Anglian adults of mean age 69 ± 2.9 years (see [2]). All analyses were conducted using R version 2.7.0 [3].

## Results

### Case

We report on a 75-year-old Caucasian woman, admitted in 1972 aged 39 for schizophrenia. She has been an informal inpatient since then. In her later years, her medical history includes atrial fibrillation, recurrent pneumonia, pulmonary fibrosis, small-vessel cerebrovascular disease, gallstones, and lupus anticoagulant without antinuclear antibody. Her regular drug treatment comprised clozapine, digoxin, furosemide, simvastatin, senna, lactulose, and zolpidem. She was on no calcium or vitamin D supplements. Aspirin had recently been replaced with warfarin, which was stopped shortly afterwards following the development of iron-deficiency anaemia. She was independently mobile and a smoker.

**Figure 1:**
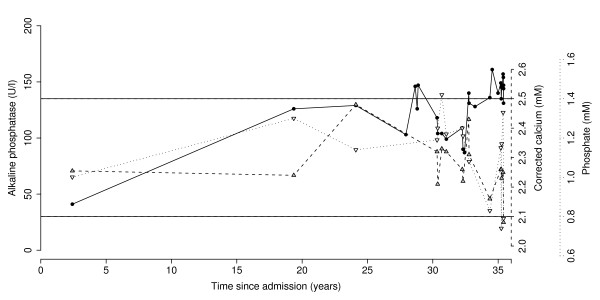
**Serum alkaline phosphatase (•), corrected calcium (▴), and phosphate (▿) for the index patient**. Horizontal lines show upper and lower limits of normal ranges for all variables. Alkaline phosphatase increased over time (linear component, *r*^2 ^= 0.46, *F*_1,28 _= 23.9, *p *= 0.000037).

**Figure 2:**
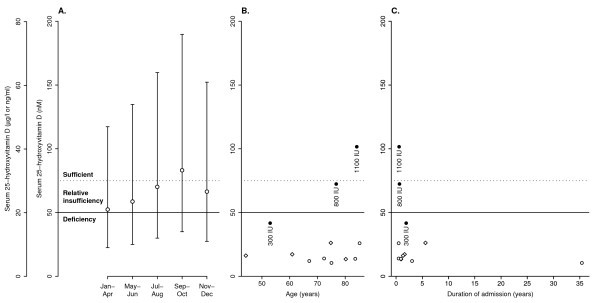
**Serum 25-hydroxyvitamin D levels in historical controls and psychiatric inpatients**. **(A) **Normal values for healthy East Anglian adults of mean age 69 years [2]. Circles show means; bars indicate 95% intervals. Solid and dotted horizontal lines indicate suggested thresholds for deficiency and sufficiency, respectively [1]; toxicity is unlikely with levels below 374 nM [1,27]. **(B, C) **Serum 25-hydroxyvitamin D levels of inpatients in the current survey (taken in April–May) shown by age, sex, duration of admission, and supplementation status. Filled symbols show patients on vitamin D supplementation, with label showing supplement amount in international units (IU) per day. Open symbols show those not supplemented. Circles show females; diamonds show males. Solid and dotted lines indicate thresholds for deficiency and sufficiency as before.

We investigated her for a mildly but persistently elevated alkaline phosphatase, with intermittently low phosphate and calcium (Figure 1). Her albumin, bilirubin, alanine aminotransferase (ALT), and renal function were normal. Bone and liver isoforms of alkaline phosphatase were not measured. The diagnosis of osteomalacia was confirmed when in April 2008 her serum 25-hydroxyvitamin D level was found to be 10.5 nM, representing severe deficiency (Figure 2A). Coeliac serology was negative. She was commenced on calcium and vitamin D supplementation but fell and suffered a subcapital fracture of her femoral neck later that night, requiring hemiarthroplasty, subsequently complicated by pneumonia, wound infection, urinary tract infection, sepsis, recurrent hemiarthroplasty dislocation and prosthesis infection requiring excision arthroplasty.

### Prevalence of vitamin D deficiency

All patients examined were Caucasian. Their mean age was 71.4 years. Eleven (92%) had suboptimal levels and 10 (83%) had frank deficiency (Figure 2B, C). Three patients were on vitamin D supplements as multivitamins, calcium plus colecalciferol tablets, or both. The best predictor of vitamin D status was supplementation (significant effect of supplementation on 25-hydroxyvitamin D levels, *F*_1,7 _= 33.7, *p *= 0.00066, in a linear model using age and admission time as continuous predictors, and supplementation and sex as two-level factors, with no interactions, and assessing the contribution of each predictor over and above that of all others). As supplementation was infrequent and heterogeneous, we analysed only the non-supplemented group further. Of those nine not on vitamin D supplements, 100% had vitamin D deficiency. There was no effect of age, sex, or duration of admission in this subgroup (*F*s < 1, NS).

In comparison to the historical control group, those patients not on supplements not only had serum 25-hydroxyvitamin D levels significantly below the seasonal control mean, but significantly below the 2.5th centile for the winter trough (*t*_8 _= 3.07, *p *= 0.015).

## Discussion

### Vitamin D deficiency and osteomalacia

Osteomalacia is defective mineralization of bone matrix (osteoid) and results from vitamin D deficiency [4,5]. Vitamin D is required for effective calcium absorption. It is absorbed from the gut as ergocalciferol (vitamin D_2_) or colecalciferol (cholecalciferol; vitamin D_3_), or made from 7-dehydrocholesterol in the skin as colecalciferol. Both vitamin D_2 _and vitamin D_3_, collectively known as vitamin D, are stored in fat. Circulating vitamin D is then hydroxylated in the liver to 25-hydroxyvitamin D, and again in the kidney (under feedback control) to 1,25-dihydroxyvitamin D, the active form [5]. Vitamin D status is best assayed by measuring serum 25-hydroxyvitamin D [1].

Few foods contain significant vitamin D. The principal dietary sources are oily fish, fortified products, and dietary supplements [1]. In the UK, vitamin D fortification is mandatory only for margarine, and milk is not usually fortified [6]. Vitamin D deficiency in the UK is very common, with 12% of Caucasian and 33% of Asian outpatients having levels < 25 nM at the end of summer [7], and 87% of middle-aged Caucasians having levels < 75 nM in winter and spring [6]. Figure 2A shows similar data for older East Anglian adults [2]. The prevalence of deficiency increases with age (as skin 7-dehydrocholesterol levels decrease), skin pigment, and obesity [1,6,7]. Lack is usually due to dietary deficiency combined with lack of sufficient sunlight-dependent synthesis in the skin. More rarely, deficiency may be due to malabsorption; liver disease; renal disease; some therapeutic drugs; hyperparathyroidism, hyperthyroidism, and granulomatous diseases (through increased vitamin D metabolism); and other rarer causes [1,4,5].

Osteomalacia causes bone pain and tenderness, skeletal deformity, and proximal muscle weakness, and predisposes to fracture [4]. Osteoporosis commonly co-exists, and osteomalacia exacerbates osteoporosis [1]. Additionally, vitamin D deficiency is associated with an increased risk of cancer, type 1 and type 2 diabetes mellitus, multiple sclerosis, Crohn's disease, hypertension, heart failure, and airways disease [1]. It is also associated with schizophrenia, perhaps via developmental vitamin D deficiency [8,9], and with depression, with some evidence for a therapeutic effect of vitamin D [10-12]. In osteomalacia, calcium and phosphate are low or normal, urinary calcium is low, and the alkaline phosphatase is typically raised as the disease progresses [4].

Patients with psychotic illnesses are at additional risk of osteoporosis, at least in part because many antipsychotic drugs cause hyperprolactinaemia that can lead to a reduction in bone mineral density [13,14]. Other endocrine mechanisms may also play a role in the high prevalence of osteoporosis in schizophrenia [15,16].

### Causes in this case

In the index case, osteomalacia was secondary jointly to inadequate dietary vitamin D intake and inadequate ultraviolet B exposure, even though she liked to be outdoors in good weather. There was likely concomitant osteoporosis. As our patient habitually ate the hospital food provided without rejection, the duration of her inpatient stay indicates that the hospital's food did not provide sufficient vitamin D over a prolonged period to prevent deficiency or perhaps to correct pre-existing deficiency. The diet currently provided is low in oily fish, and efforts are underway to improve it.

### High prevalence of vitamin D deficiency

This was a small survey. Nonetheless, it is clear that this population is at extremely high risk of vitamin D deficiency, and in our results this was independent of the duration of admission. Vitamin D deficiency is common in the UK [6,7], yet the prevalence and degree of vitamin D deficiency in our study population was significantly higher than in healthy historical controls of a similar age.

### Suggestions

Osteomalacia must always be considered in the differential diagnosis of a raised alkaline phosphatase. We suggest that all hospitals should undergo regular review by a dietician to ensure adequate vitamin D is provided or supplementation is in place; this is particularly important for environments where patients are resident for prolonged periods, such as long-stay psychiatric units. Psychiatrists should ensure vitamin D supplementation is adequate. Vitamin D level screening is worthwhile in high-risk populations, and supplementation can reduce the risk of fracture in the elderly, and a range of other major diseases [1]. In addition to effects on bone metabolism, there is evidence from meta-analysis of randomized controlled trials that vitamin D supplementation can reduce the risk of falls in the elderly [17,18]; the mechanism may be via lower limb muscle strength and improved balance [19-21].

### Prevention and treatment of simple deficiency

A daily intake of 800–1,000 international units (IU) (20–25 μg) is probably desirable to prevent deficiency in adults without other major risk factors; achieving this may require widespread supplementation [1]. More may be required in the obese, in pregnant and lactating women, and if other risk factors for deficiency exist [1]. The European Community Recommended Daily Amount (EC RDA) is only 200 IU [22], which is inadequate [1]. Vitamin D_2 _and vitamin D_3 _are both effective supplements [23]; suggestions for maintenance supplementation are shown in Table 1. Treatment of established deficiency requires higher doses of vitamin D. One strategy for treating deficiency due to dietary or sunlight deficiency is to give 50,000 IU of ergocalciferol orally once weekly for 8 weeks, repeating for another 8 weeks if vitamin D levels remain < 75 nM [1]. Another simple regimen is to give ergocalciferol 10,000 IU orally once daily during this treatment period [24], or intramuscular colecalciferol 300,000 IU monthly [24]. Vitamin D deficiency secondary to more complex causes or when renal failure is present may need still higher doses or, if vitamin D metabolism is significantly impaired, calcitriol (1,25-hydroxycolecalciferol) [25]. The need for simultaneous calcium supplementation, to achieve a total daily intake of the order of 1,000–1,200 mg, should always be considered [26].

**Table 1:** Examples of regimes for treating simple vitamin D deficiency (deficiency secondary to inadequate sunlight exposure or dietary intake), and maintenance supplementation.

**Regime**	**Approximate daily dose**	**Comments and disadvantages**
**Treatment of vitamin D deficiency**		

ergocalciferol 250 μg (10,000 IU) once daily	10,000 IU vitamin D	Supply problem in the UK.

ergocalciferol 1.25 mg (50,000 IU) once weekly	7,000 IU vitamin D	Supply problem in the UK.

intramuscular ergocalciferol or colecalciferol 7.5 mg (300,000 IU) monthly	10,000 IU vitamin D	Injection may be unpopular. Supply problem in the UK.

paediatric ergocalciferol solution (3,000 IU/ml), 3 ml daily	9,000 IU vitamin D	Special supply arrangements may be required. Excipients may include peanut oil.

colecalciferol 500 μg (20,000 IU) 3–4 times per week	8,600–11,400 IU vitamin D	Available from overseas suppliers.

colecalciferol liquid, e.g. 2,000 IU/ml, 5 ml daily	10,000 IU vitamin D	Custom strengths available as 'special' orders in the UK.

commercial 'high strength' (25 μg; 1,000 IU) colecalciferol, two tablets twice daily	4,000 IU vitamin D	Available from high street health food suppliers including online.

colecalciferol 25 μg (1,000 IU), two tablets twice daily, *plus *compound calcium (500 mg) with ergocalciferol or colecalciferol (400 IU), one tablet twice daily	4,800 IU vitamin D 1,000 mg calcium	Includes sufficient calcium to ensure adequate total intake (though calcium supplements may have low palatability).

compound calcium (500 mg) with ergocalciferol or colecalciferol (400 IU), two tablets twice daily, *plus *generic multivitamins (including 300 IU vitamin D), two capsules twice daily	2,800 IU vitamin D 2,000 mg calcium 10,000 IU vitamin A (3,000 μg RE) 60 mg ascorbic acid 30 mg nicotinamide 2 mg riboflavin 4 mg thiamine	Not ideal. Vitamin D dose lower than recommended treatment regimens. Many tablets required. Calcium content gives poor palatability and may cause gastrointestinal side effects in a few patients [37]. The vitamin A dose may be close to a prudent threshold for teratogenicity, and exceeds the maximum dose recommended for long-term use (1,500 μg RE/day); higher doses are associated with increased risk of hip fracture [37]. No other component is likely to produce toxicity at this dose [37].

1-hydroxylated derivatives of vitamin D (e.g. alfacalcidol, dihydrotachysterol, calcitriol)	--	Not recommended. Does not treat vitamin D deficiency. Higher potential for toxicity. Greater need for monitoring. Few reasons to use, unless severe renal disease or hypocalcaemia [31-34].

**Maintenance supplementation (examples)**		

ergocalciferol 1.25 mg (50,000 IU) every 2–4 weeks or monthly	1,640–3,570 IU vitamin D	Supply problem in the UK.

colecalciferol 25 μg (1,000 IU) daily	1,000 IU vitamin D	--

compound calcium (500 mg) with ergocalciferol or colecalciferol (400 IU), one tablet twice daily	800 IU vitamin D 1,000 mg calcium	Includes sufficient calcium to ensure adequate total intake.

**Additional non-pharmacological options**		

sunlight exposure (0.5 minimal erythemal dose daily, e.g. 5–10 minutes of exposure of arms and legs to direct sunlight)	3,000 IU vitamin D [1]	Excessive exposure predisposes to skin cancer. Latitude, season, time of day and weather alter incoming radiation dose; age and skin pigment alter efficacy.

cod liver oil (5 ml/day)	400–1,000 IU vitamin D [1] 2,400–15,600 IU vitamin A (720–4,700 μg RE) [38] other constituents including Ω-3 fatty acids	Caution advised in asthma and pregnancy and in patients on warfarin [39]. May convey other health benefits. Some preparations can contain high doses of vitamin A, which may be disadvantageous (see above).

oily fish (two portions of 100 g per week)	70–285 IU vitamin D [1] other constituents including Ω-3 fatty acids	May convey other health benefits.

Suboptimal regimes are included for illustration. Note that more may be required in pregnant and lactating women and the obese, and if other risk factors for deficiency exist. Recheck vitamin D levels after every 8 weeks of treatment and return to a maintenance regime when serum 25-hydroxyvitamin D levels are satisfactory (> 75 nM). Always consider the need for supplemental calcium to achieve a total daily intake of 1,000–1,200 mg [1]. All treatments are oral unless stated. (IU, international units; RE, retinol equivalents of vitamin A.)

### Risk of vitamin D toxicity

Vitamin D toxicity is unlikely to occur with serum 25-hydroxyvitamin D levels below 374 nM unless there is hypersensitivity to vitamin D (e.g. hyperparathyroidism, granulomatous diseases) [1,27]. Vitamin D toxicity is caused by hypercalcaemia [27] and when very high doses of vitamin D are used or if there is renal impairment, plasma calcium monitoring is required weekly and if nausea or vomiting occurs [25]. Thiazides and related diuretics also increase the risk of hypercalcaemia [25]. However, doses of 10,000 IU per day are equivalent to what may be obtained from sunlight and are extremely unlikely to cause toxicity [1,27,28].

### Treatment options given vitamin D supply problems in the UK

A significant practical problem is that there is currently a supply failure of high-dose oral and injectable ergocalciferol in the UK [29]. Colecalciferol is not listed in a high-dose (> 800 IU/day) preparation in the British National Formulary [25]. The 1-hydroxylated derivatives of vitamin D available (alfacalcidol, dihydrotachysterol, and calcitriol) are much more potent, escape a key metabolic regulatory step, and have a rapid onset and offset of action as they are not stored in significant quantities in fat [1,5]. Thus, although they are biologically active whilst they are being given, they do not replenish bodily stores of vitamin D [30]. They are also more likely to cause hypercalcaemia, and require calcium monitoring. They are not recommended for treating simple vitamin D deficiency without hypocalcaemia [31-34]. Alternatives strategies for restoration of normal bodily stores therefore include high doses of compound preparations [35], paediatric formulations, and commercial supplements. Practical approaches to this problem are outlined in Table 1.

## Conclusion

This study illustrates the high prevalence of vitamin D deficiency in an old-age psychiatric rehabilitation unit, something also observed recently but to a lesser extent in a younger psychiatric inpatient population [36]. Vitamin D deficiency is extremely common, especially in countries far from the equator and where food supplementation is not widespread. It predisposes to a wide range of serious diseases. Key points are that (1) the serum 25-hydroxyvitamin D level is the best measure of vitamin D status; (2) many patients in geriatric psychiatry units will need treatment for vitamin D deficiency, and most need maintenance supplementation; and (3) supply problems currently complicate the prescription of high-dose vitamin D in the United Kingdom.

## Competing interests

The authors declare that they have no competing interests.

## Authors' contributions

RNC collected and analysed the data, wrote the first draft, and serves as guarantor; both authors edited the manuscript.
